# Stenosis of the Larynx

**Published:** 1909-03-13

**Authors:** 


### Laryngology and Rhinology.

### STENOSIS OF THE LARYNX.

Narrowing of the lumen of the larynx may be produced by many forms of disease, which may for convenience be grouped under the following heads:

1. *Inflammatory Swelling.—Œdematous Laryngitis.*—In adults, stenosis as the result of simple inflammation is very rare, but occurs with the severer inflammatory affections, such as phlegmonous laryngitis; or as the consequence of traumatism, such as the inhalation of steam or corrosive fluids. Inflammation spreading from without may produce œdema, as in angina Ludovici. In children, a simple laryngitis may proceed to œdema sufficiently marked to cause asphyxiation; and severe œdema is produced by scalds of the throat, which result from attempting to drink from the spout of a kettle or some similar misadventure. The œdema is most marked at the upper aperture of the larynx, especially about the arytenoids and aryteno-epiglottidean folds; it is therefore inaccurately described as "œdema of the glottis." Under this heading, also, may be included perichondritis of the laryngeal cartilages; this affection is most frequently the result of secondary infection with pyogenic micro-organisms during the course of tuberculous, syphilitic, or malignant disease, but it also occurs as a complication of enteric fever, smallpox, or other exanthema, and it occasionally results from injury.

2. *The Granulomata.*—Syphilis is a commoner cause of laryngeal stenosis than any other disease, but it is generally the subsequent cicatricial contraction rather than the actual infiltration which is the cause of the narrowing; but a gumma sometimes produces decided obstruction. Tuberculous laryngitis occasionally, though very rarely, causes enough narrowing to produce dyspnœa, and it seldom results in cicatrisation sufficiently marked to give rise to stenosis. In lupus of the larynx the infiltration is not massive enough to obstruct the lumen, but the subsequent contraction may readily do so. Leprosy, glanders, and scleroma may also produce stenosis.

3. *Neoplasms.*—Malignant growths, especially epitheliomata, may, of course, block the lumen. Of innocent tumours the only examples which obstruct the larynx at all frequently are multiple papillomata in children; in adults papillomata are less often multiple, and are nearly always removed long before they reach an obstructive size. Rare instances have been recorded of other neoplasms, such as fibromata and lipomata, which have attained enormous dimensions in the larynx and caused stenosis; but it is extraordinary to what extent the lumen of the larynx may become blocked without producing any very marked dyspnœa, provided only that the origin and progress of the obstruction is slow and gradual.

4. *Foreign Bodies.*—These may completely block the upper aperture or the lumen of the larynx and cause rapid asphyxiation; but if a body of small size becomes impacted in the larynx the resulting dyspnœa is due to spasm, which generally soon subsides, though it may recur or be followed by inflammatory swelling.

5. *Paralysis and Fixation of the Vocal Cord in the Adducted Position.*—These affections, when unilateral, do not cause much dyspnœa except on exertion; in children, however, the dyspnœa may be very marked. Bilateral abductor paralysis is a cause of very severe stenosis, and necessitates the permanent wearing of a tracheotomy tube; it is sometimes caused by pressure on both recurrent laryngeal nerves, but it is more usually of central origin and due to tabes or pachymeningitis at the base of the brain.

6. *Spasm of the Glottis.*—The causes of glottic spasm are very numerous, and cannot be thoroughly discussed here; they produce, of course, only a temporary stenosis, but the importance of spasm in this connection is that it very often increases the stenosis due to other lesions, especially in young people, and it is this factor which imparts the intermittent character to the dyspnœa which is so frequently observed in cases of organic stenosis.

7. *Cicatricial Stenosis.*—The formation of scar-tissue is of all the most important form of stenosis. It may result from any kind of trauma, and is not very uncommon after thyrotomy, especially if granulations are allowed to form in the anterior commissure, or if the sutures are passed right through the cartilage into the lumen of the larynx. Cicatricial stenosis results after any severe ulceration or deep inflammation, and is said to be very severe after typhoid perichondritis, but a very large majority of cases are the result of syphilis. The narrowing is usually in the neighbourhood of the glottis, and most marked at the anterior commissure; the cords often become adherent in front, or a web may form between them, but the cartilaginous glottis usually remains free. A web may be thin or very thick and solid, or the stenosis may be deep, extend into the trachea and reduce the lumen to a narrow tube. The arytenoid cartilages, also, may be fixed by scar-tissue in an adducted position.

8. *Congenital Abnormalities.*—Congenital laryngeal stridor and congenital web-formations may produce congenital stenosis.

9. *Non-inflammatory Œdema.*—This is a decidedly uncommon form of stenosis, caused by renal disease or cardiac disease or angeio-neurotic œdema. The first may occur in acute or chronic renal disease and produce sudden and fatal asphyxia.

(*To be continued.*)

